# Supplementary material for: Morphometrics Reveals Complex and Heritable Apple Leaf Shapes
Source: Front Plant Sci. 2018 Jan 4;8:2185. doi: 10.3389/fpls.2017.02185 (PMC5758599; doi:10.3389/fpls.2017.02185)
Supplement: Figure S1 — Distribution of leaf phenotypes following REML-adjustment. N is equal to the total number of unique samples. [file Image1.PDF]

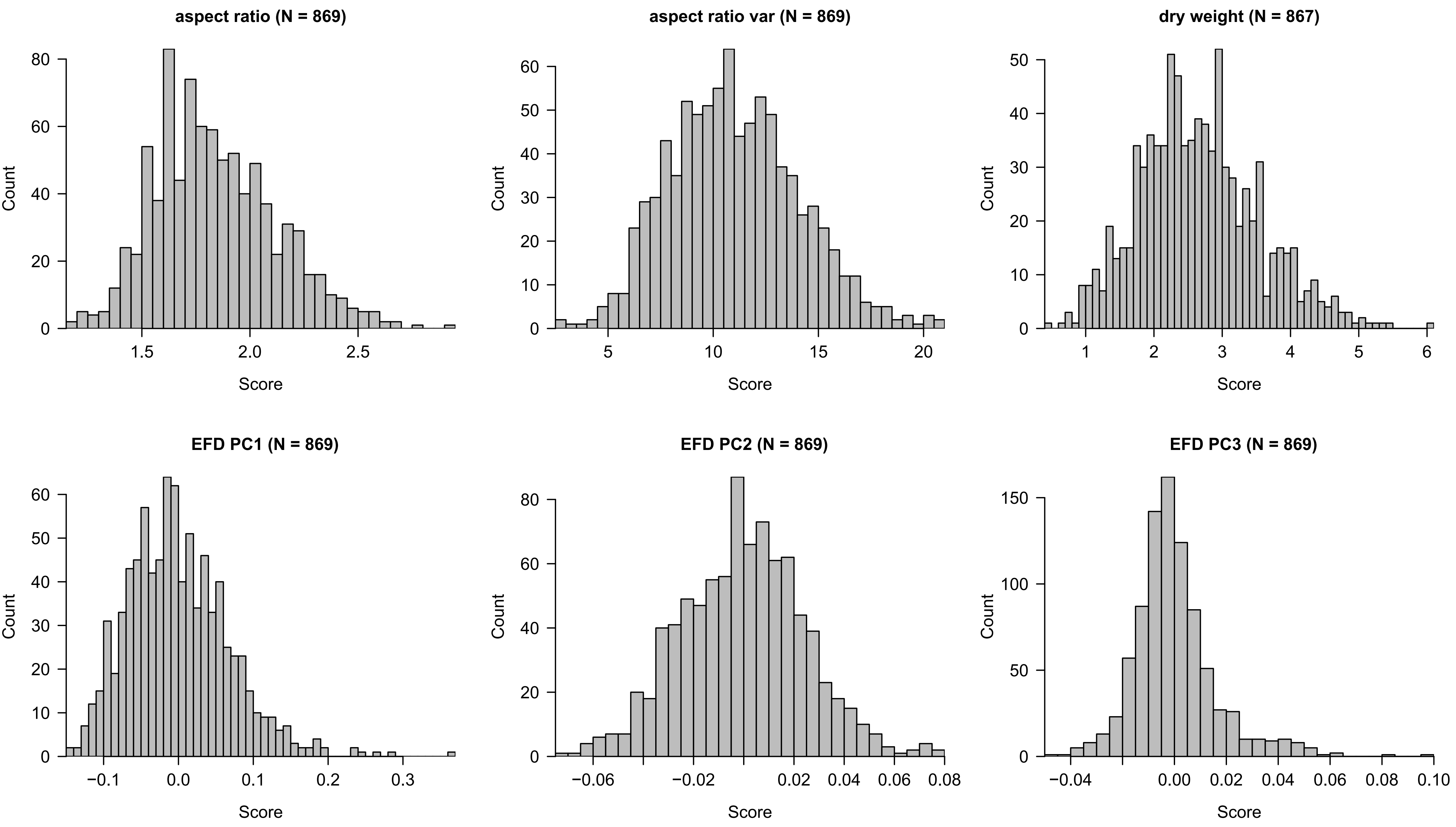

aspect ratio (N = 869)
aspect ratio var (N = 869)
dry weight (N = 867)
EFD PC1 (N = 869)
EFD PC2 (N = 869)
EFD PC3 (N = 869)
Count
Score

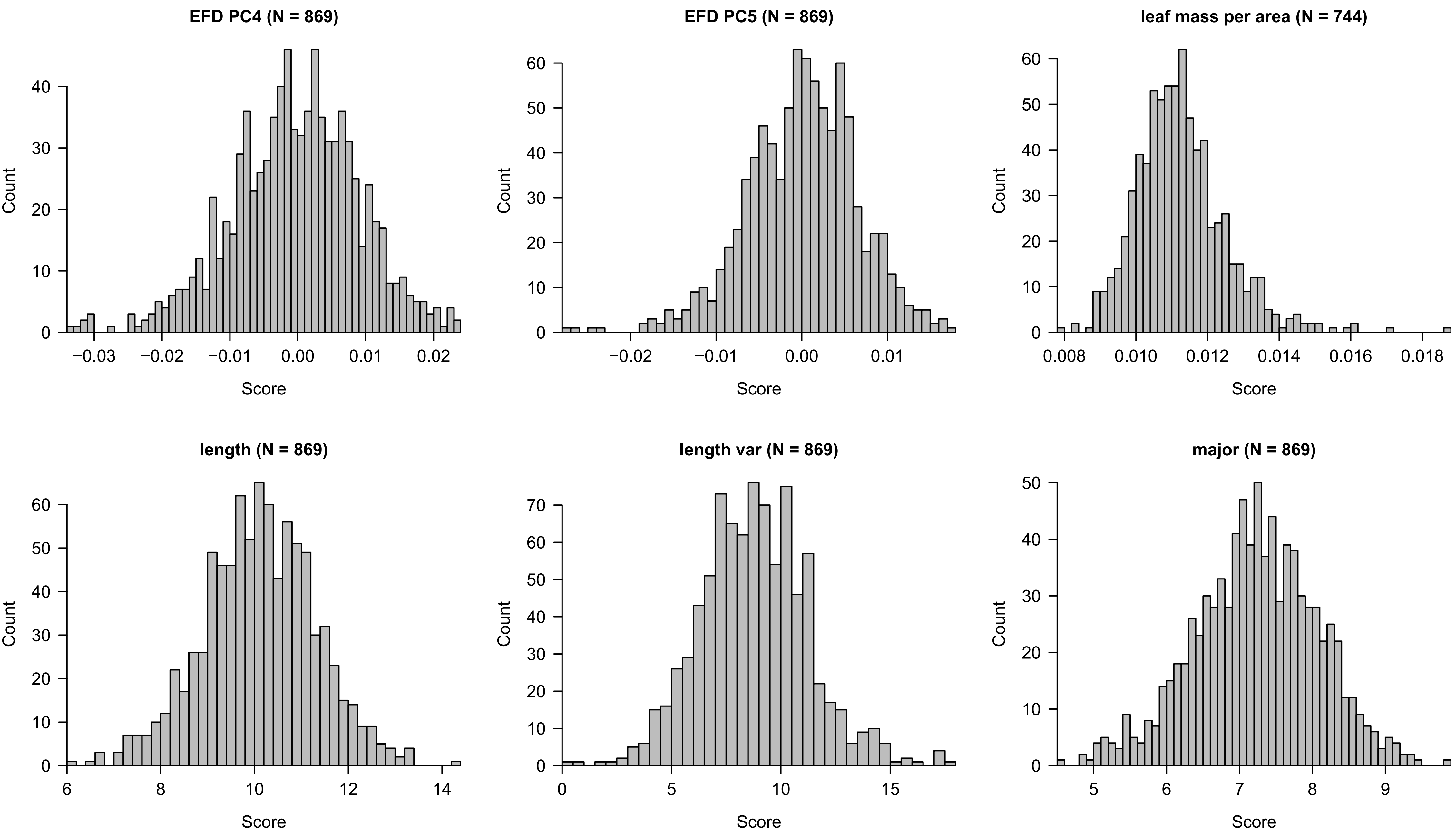
EFD PC4 (N = 869)
EFD PC5 (N = 869)
leaf mass per area (N = 744)
length (N = 869)
length var (N = 869)
major (N = 869)
Count
Score
−0.03 −0.02 −0.01 0.00 0.01 0.02
−0.02 −0.01 0.00 0.01
0.008 0.010 0.012 0.014 0.016 0.018
6 8 10 12 14
0 5 10 15
5 6 7 8 9
0 10 20 30 40
0 10 20 30 40 50 60
0 10 20 30 40 50 60 70

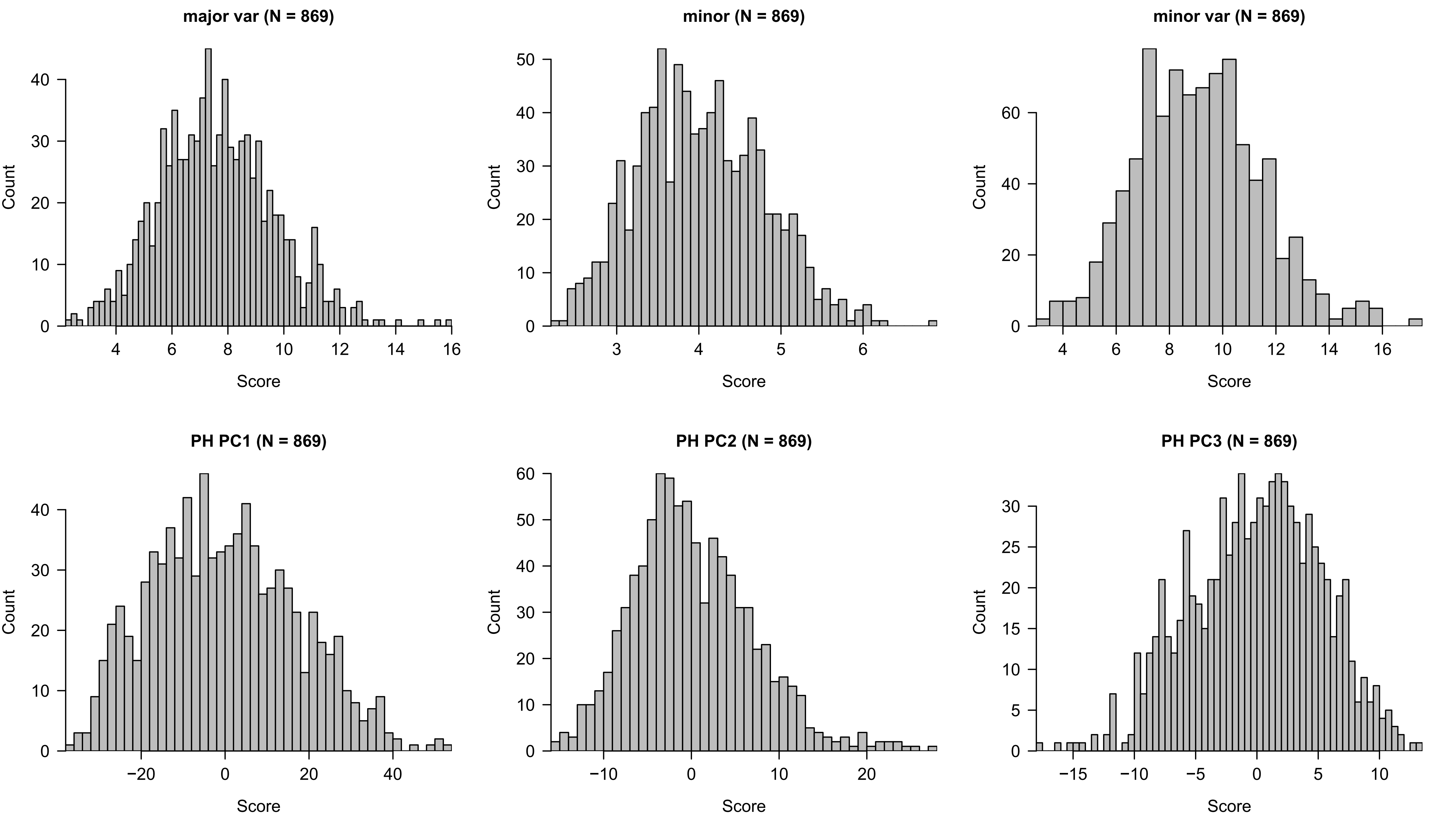
major var (N = 869)
minor (N = 869)
minor var (N = 869)
PH PC1 (N = 869)
PH PC2 (N = 869)
PH PC3 (N = 869)
Count
Score

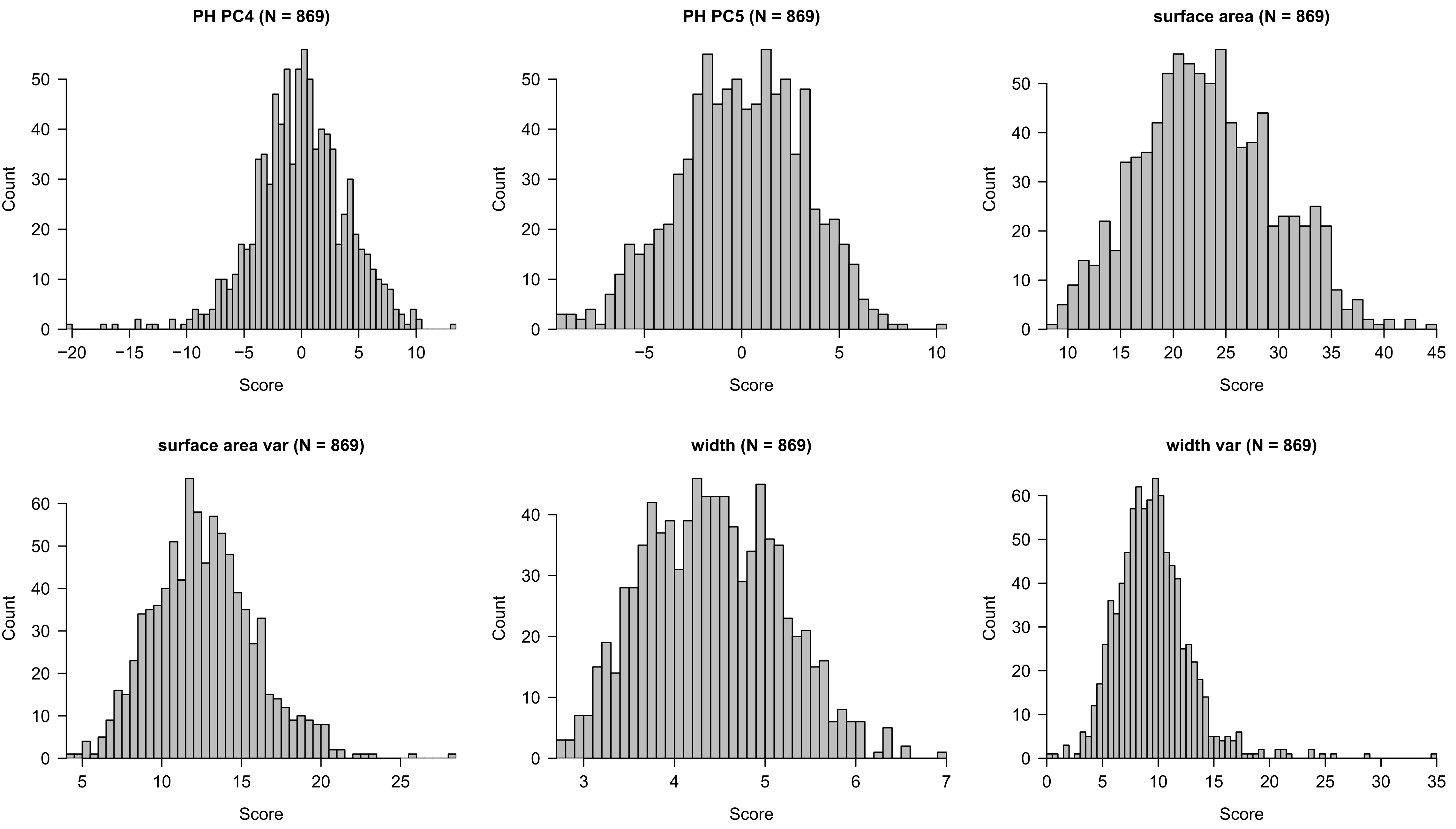
PH PC4 (N = 869)
PH PC5 (N = 869)
surface area (N = 869)
surface area var (N = 869)
width (N = 869)
width var (N = 869)
Count
Score
−20 −15 −10 −5 0 5 10
−5 0 5 10
10 15 20 25 30 35 40 45
5 10 15 20 25
3 4 5 6 7
0 5 10 15 20 25 30 35
0 10 20 30 40 50 60
