# Supplementary figures and images for: Morphometrics Reveals Complex and Heritable Apple Leaf Shapes
Source: Front Plant Sci. 2018 Jan 4;8:2185. doi: 10.3389/fpls.2017.02185 (PMC5758599; doi:10.3389/fpls.2017.02185)

**a****min PH PC1****max PH PC1**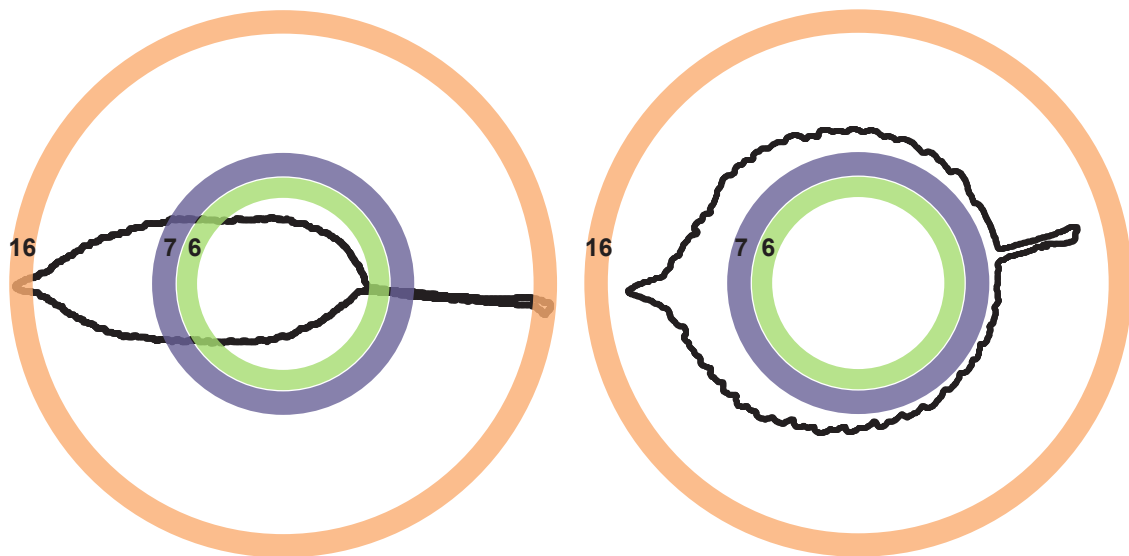**b**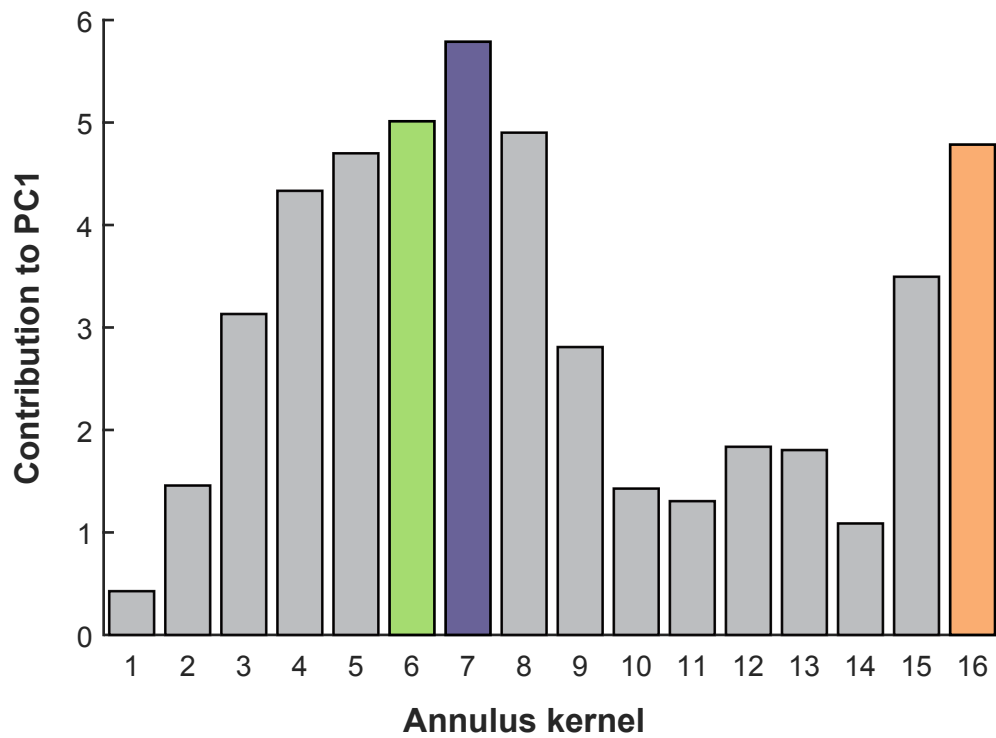

Supplement: Figure S2 — Visualization of contributions of each annulus kernel to PH PC1. Annulus kernels 6, 7 and 16 contribute the most to leaf shape according to PH PC1. The placement of each annulus kernel is visualized on a leaf representing the minimum and maximum value along PC1 (A). The contribution to PC1 of each of the 16 annulus kernels is also shown (B). [file Image2.PDF]

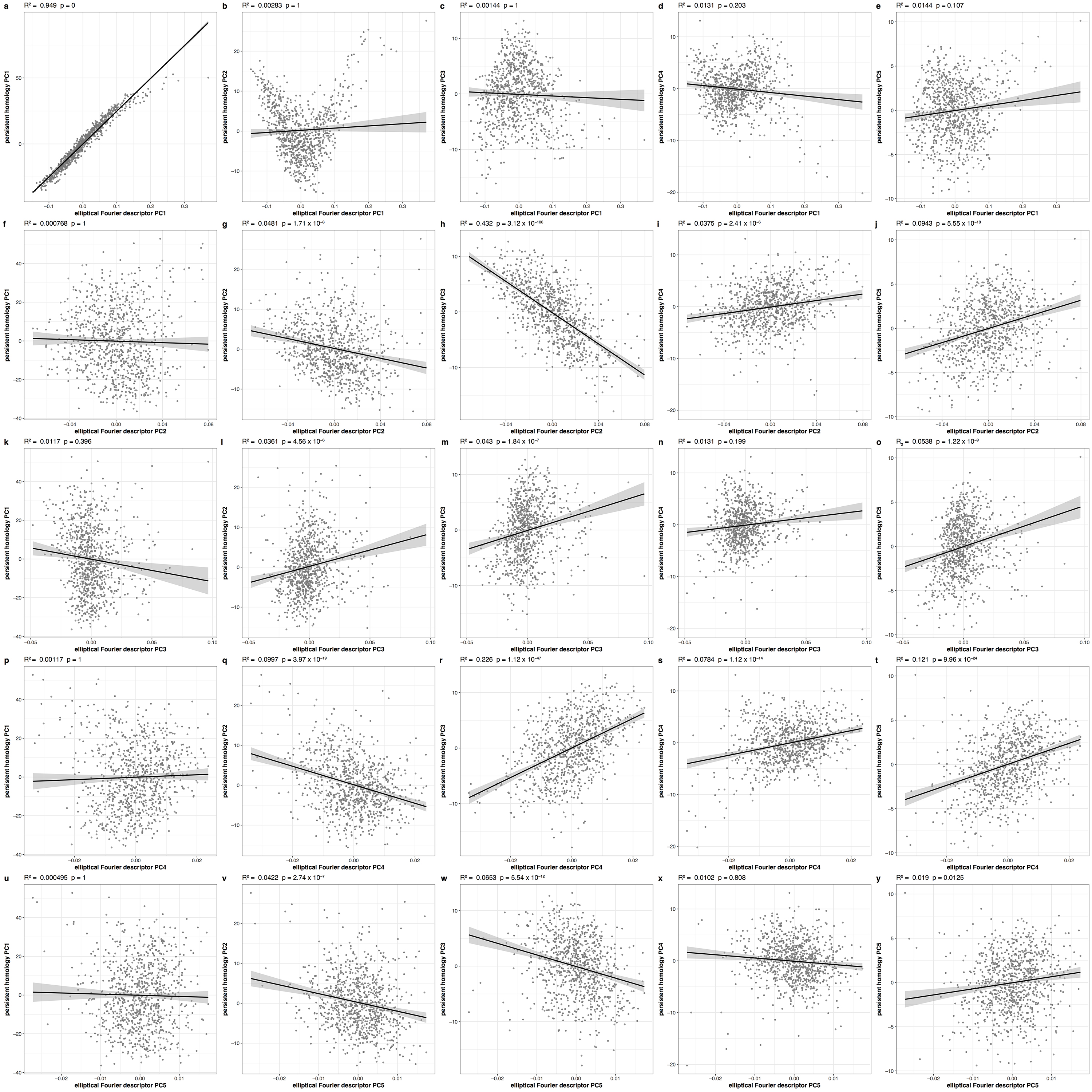

Supplement: Figure S3 — Comparison of morphometric EFD and PH PCs 1 to 5. Correlation between first 5 PCs, estimated using Pearson's correlation, including R2 and Bonferroni corrected p-values based on Figure 4, Table S3. [file Image3.JPEG]

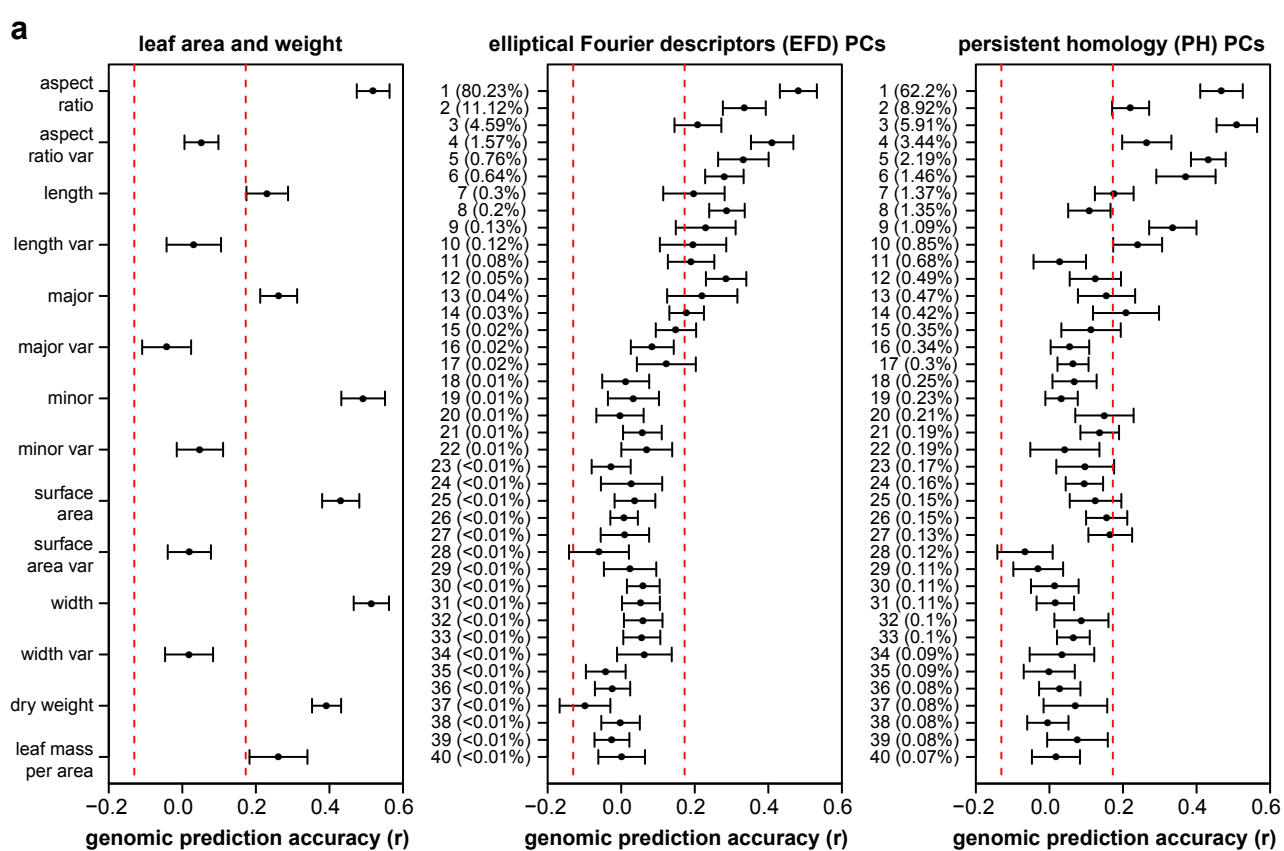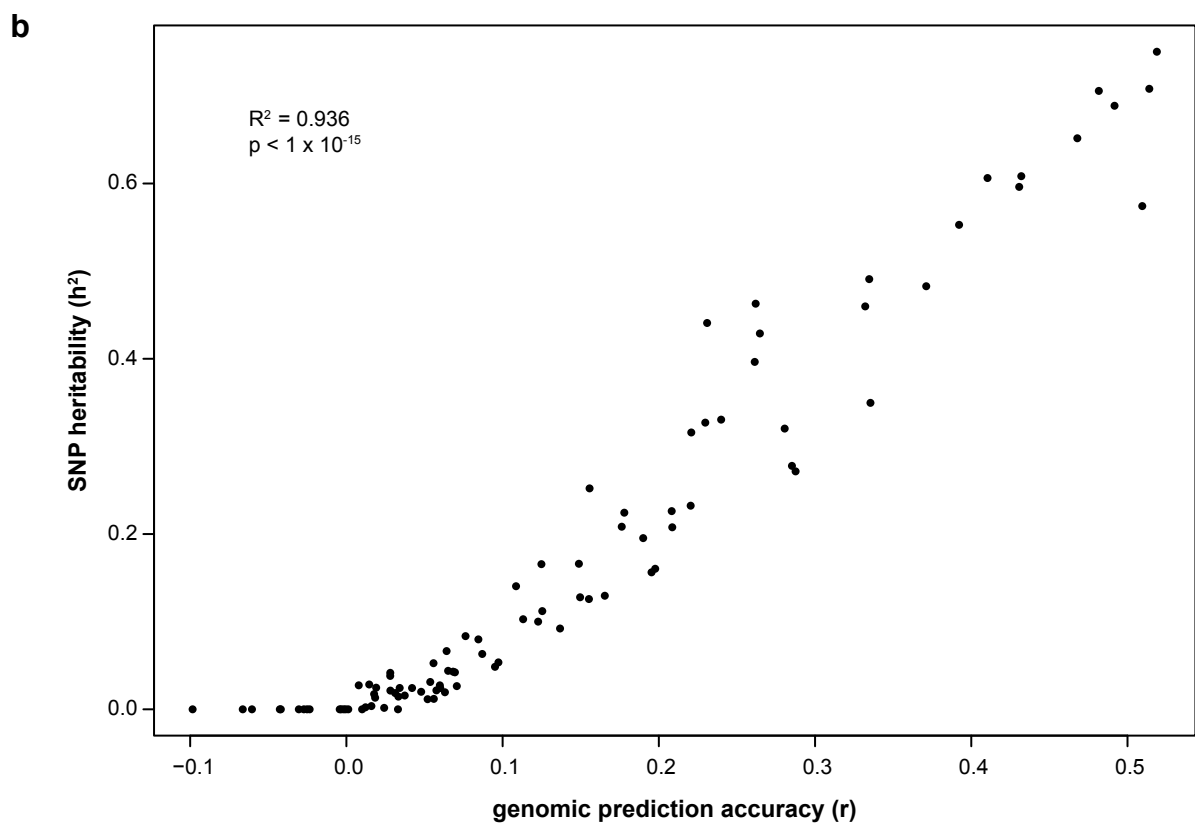

Supplement: Figure S5 — Genomic prediction accuracy (r) (A) and correlation between genomic prediction results and SNP heritability estimates (h2) for all leaf phenotypes (B). Genomic prediction accuracies represent the average correlation (± standard deviation) between observed and predicted phenotype scores, based on 5-fold cross-validation with 3 iterations. Dotted red lines indicate the minimum and maximum prediction average accuracy (r) achieved using 1,000 randomly generated phenotypes. The percent variance explained by each PC was calculated prior to REML-adjustment and is indicated in parentheses. [file Image5.PDF]
