# Supplementary material for: Morphometrics Reveals Complex and Heritable Apple Leaf Shapes
Source: Front Plant Sci. 2018 Jan 4;8:2185. doi: 10.3389/fpls.2017.02185 (PMC5758599; doi:10.3389/fpls.2017.02185)
Supplement: Figure S4 — GWAS results for all 24 leaf phenotypes examined. Manhattan and QQ plots are included for each phenotype. The QQ-plot shows both the results of a naive GWAS (Pearson correlation) and the results from applying the mixed model. P-values are log-transformed and the threshold for significance is simpleM-corrected and indicated by a horizontal dotted line. Chromosome R indicates SNPs found on contigs unanchored to the reference genome. [file Image4.PDF]

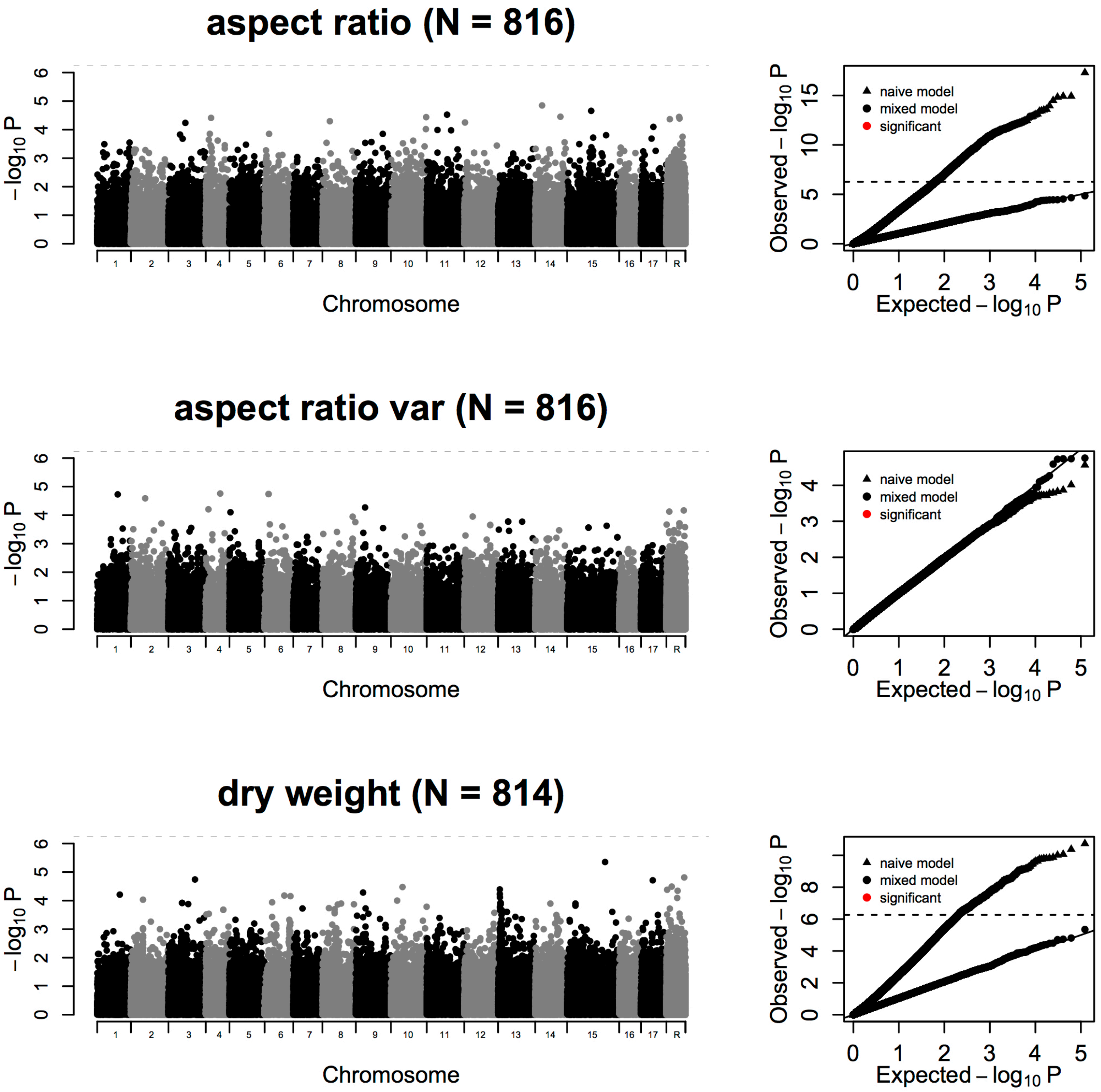
aspect ratio (N = 816)
$-\log_{10} P$
Chromosome
1 2 3 4 5 6 7 8 9 10 11 12 13 14 15 16 17 R
naive model
mixed model
significant
Observed $-\log_{10}$ P
Expected $-\log_{10}$ P
aspect ratio var (N = 816)
$-\log_{10} P$
Chromosome
1 2 3 4 5 6 7 8 9 10 11 12 13 14 15 16 17 R
naive model
mixed model
significant
Observed $-\log_{10}$ P
Expected $-\log_{10}$ P
dry weight (N = 814)
$-\log_{10} P$
Chromosome
1 2 3 4 5 6 7 8 9 10 11 12 13 14 15 16 17 R
naive model
mixed model
significant
Observed $-\log_{10}$ P
Expected $-\log_{10}$ P

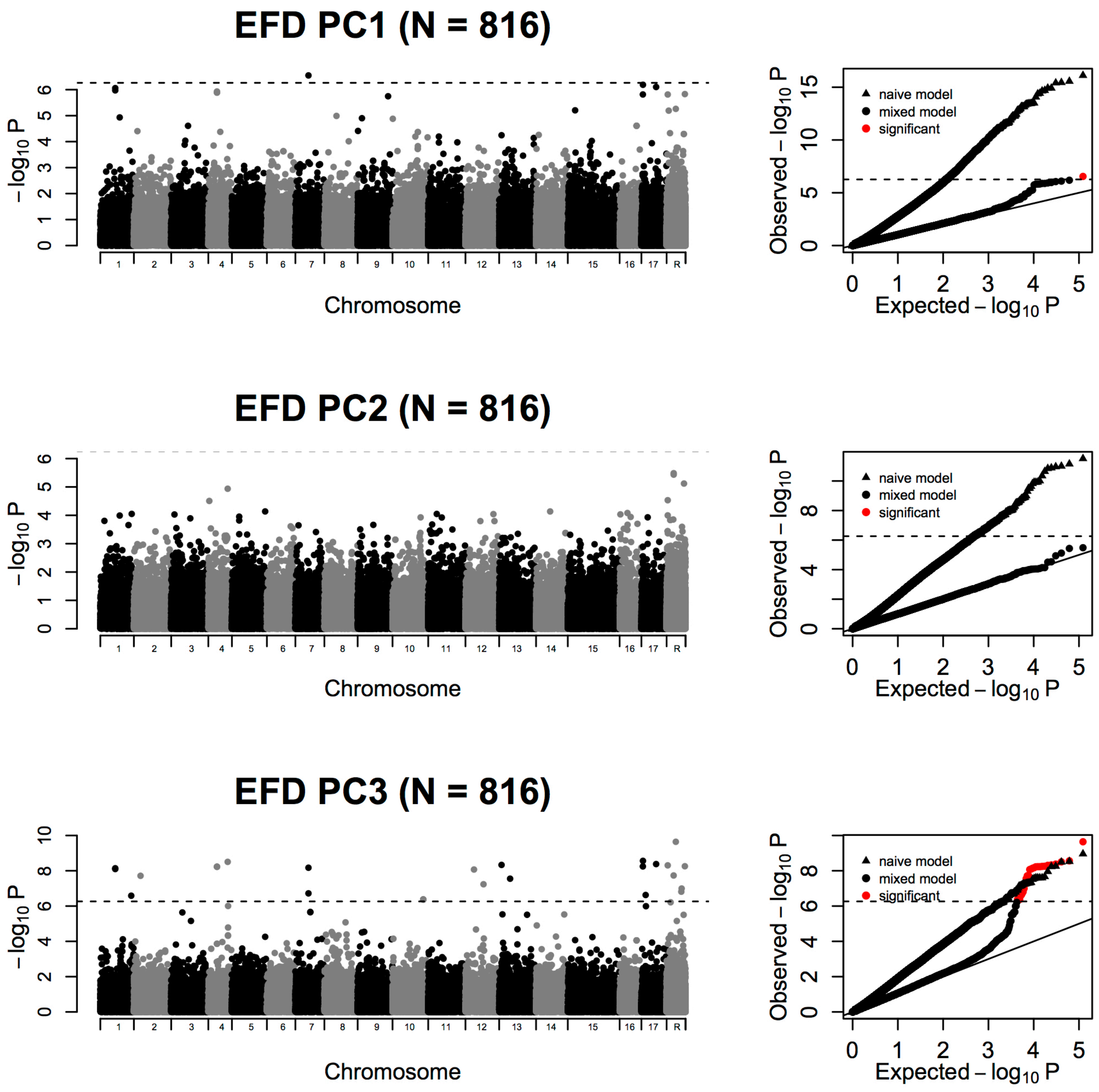
EFD PC1 (N = 816)
− log10 P
Chromosome
1 2 3 4 5 6 7 8 9 10 11 12 13 14 15 16 17 R
Observed − log10 P
Expected − log10 P
naive model
mixed model
significant
EFD PC2 (N = 816)
− log10 P
Chromosome
1 2 3 4 5 6 7 8 9 10 11 12 13 14 15 16 17 R
Observed − log10 P
Expected − log10 P
naive model
mixed model
significant
EFD PC3 (N = 816)
− log10 P
Chromosome
1 2 3 4 5 6 7 8 9 10 11 12 13 14 15 16 17 R
Observed − log10 P
Expected − log10 P
naive model
mixed model
significant

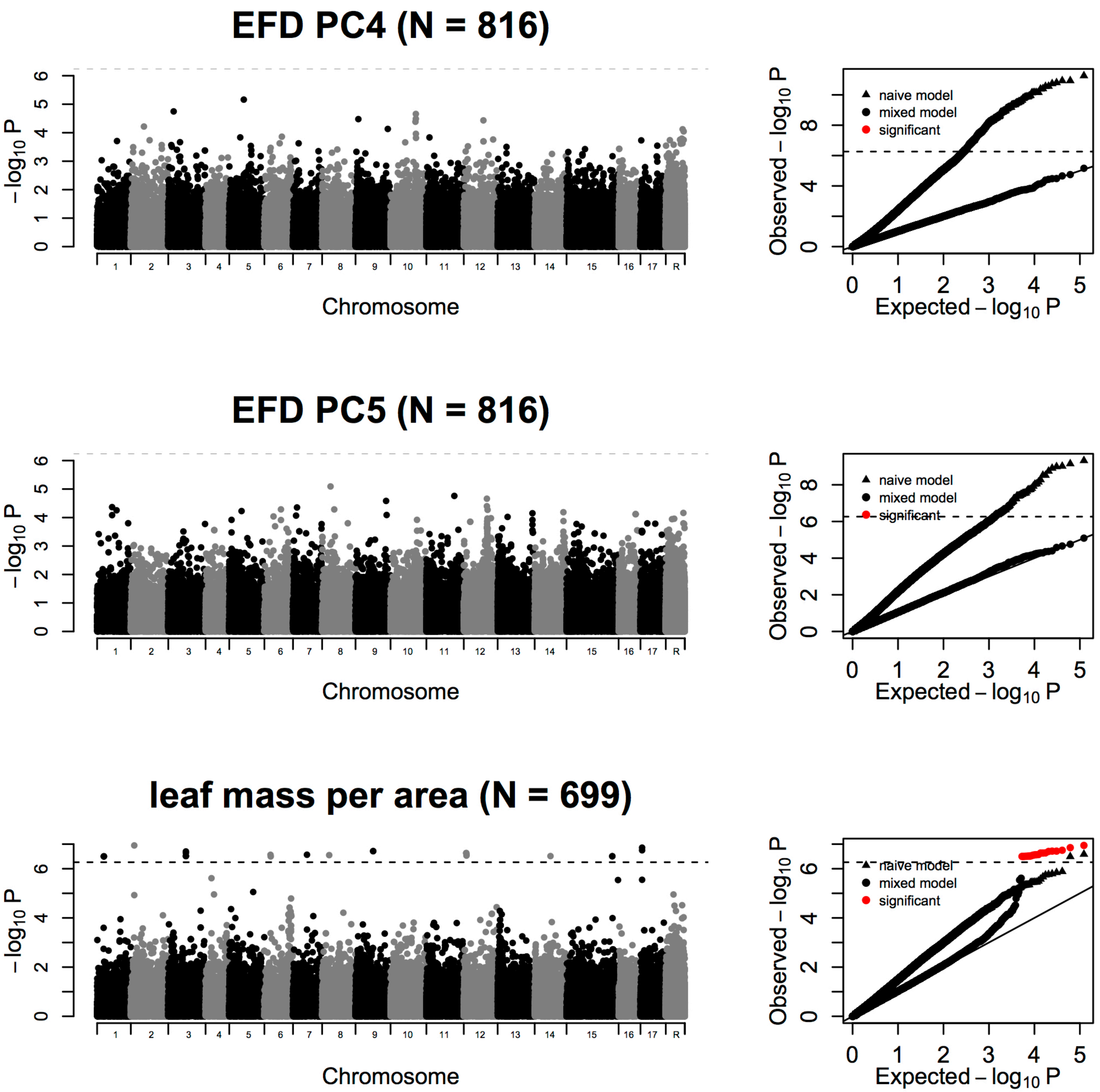
EFD PC4 (N = 816)
− log10 P
Chromosome
1 2 3 4 5 6 7 8 9 10 11 12 13 14 15 16 17 R
naive model
mixed model
significant
Observed − log10 P
Expected − log10 P
EFD PC5 (N = 816)
− log10 P
Chromosome
naive model
mixed model
significant
Observed − log10 P
Expected − log10 P
leaf mass per area (N = 699)
− log10 P
Chromosome
naive model
mixed model
significant
Observed − log10 P
Expected − log10 P

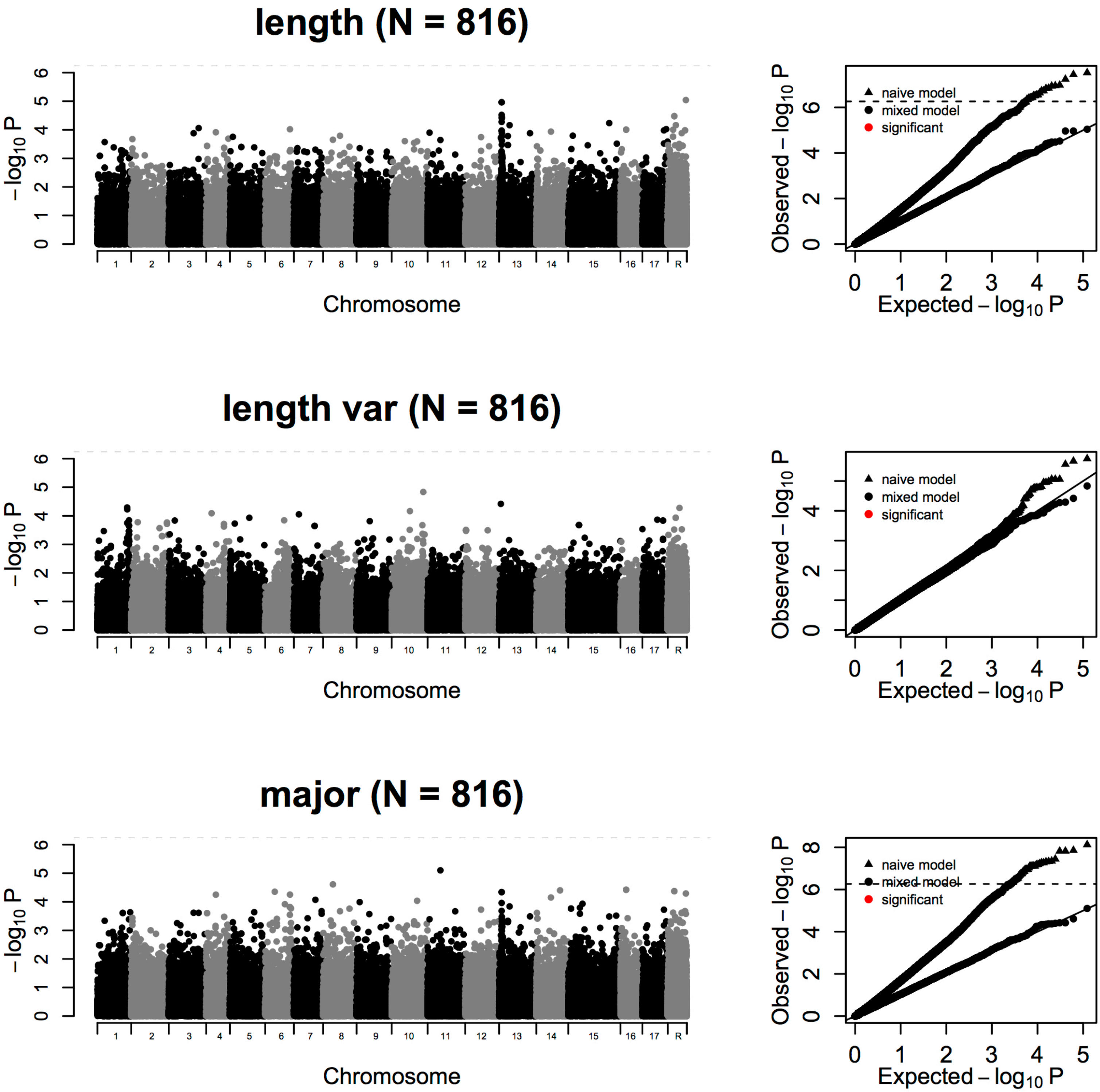
length (N = 816)
length var (N = 816)
major (N = 816)
−log10 P
Chromosome
1 2 3 4 5 6 7 8 9 10 11 12 13 14 15 16 17 R
Observed − log10 P
Expected − log10 P
naive model
mixed model
significant

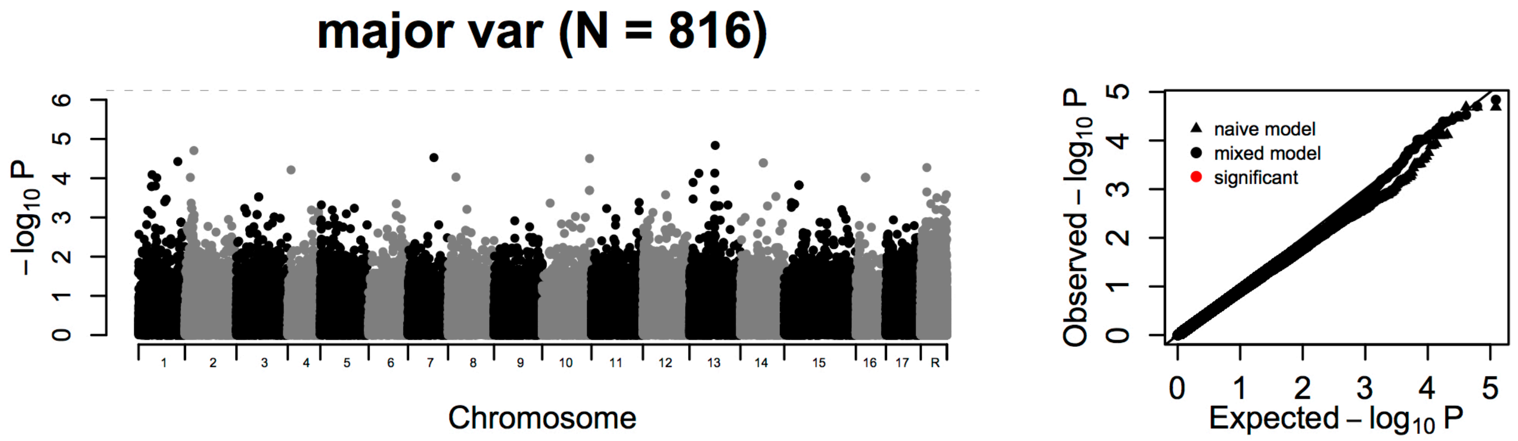

major var (N = 816)
−log10 P
0 1 2 3 4 5 6
1 2 3 4 5 6 7 8 9 10 11 12 13 14 15 16 17 R
Chromosome
Observed − log10 P
Expected − log10 P
naive model
mixed model
significant

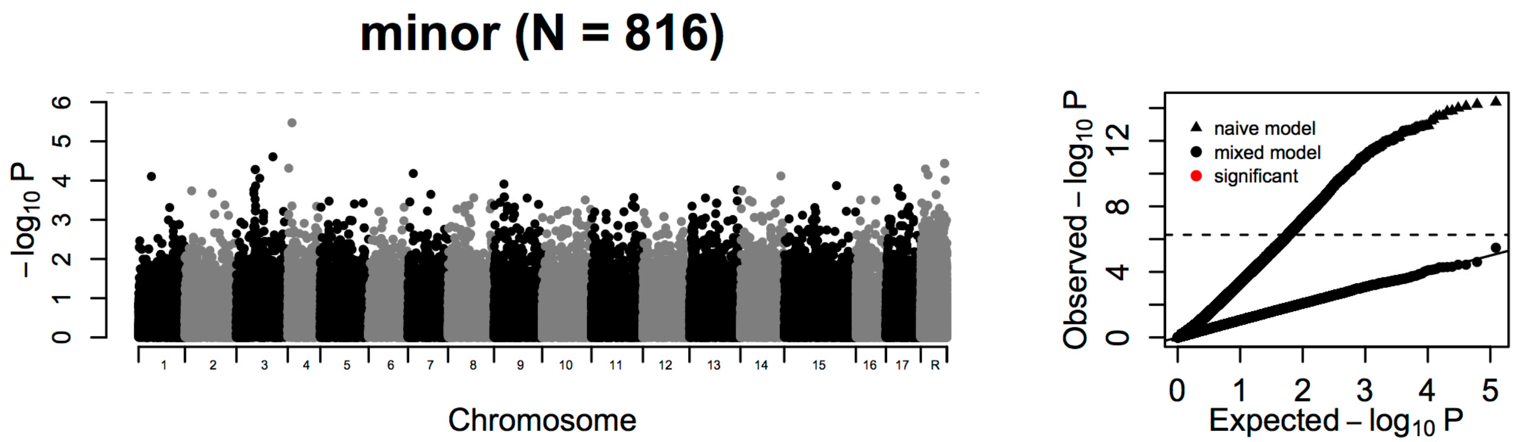

minor (N = 816)
−log10 P
0 1 2 3 4 5 6
1 2 3 4 5 6 7 8 9 10 11 12 13 14 15 16 17 R
Chromosome
Observed − log10 P
0 4 8 12
Expected − log10 P
naive model
mixed model
significant

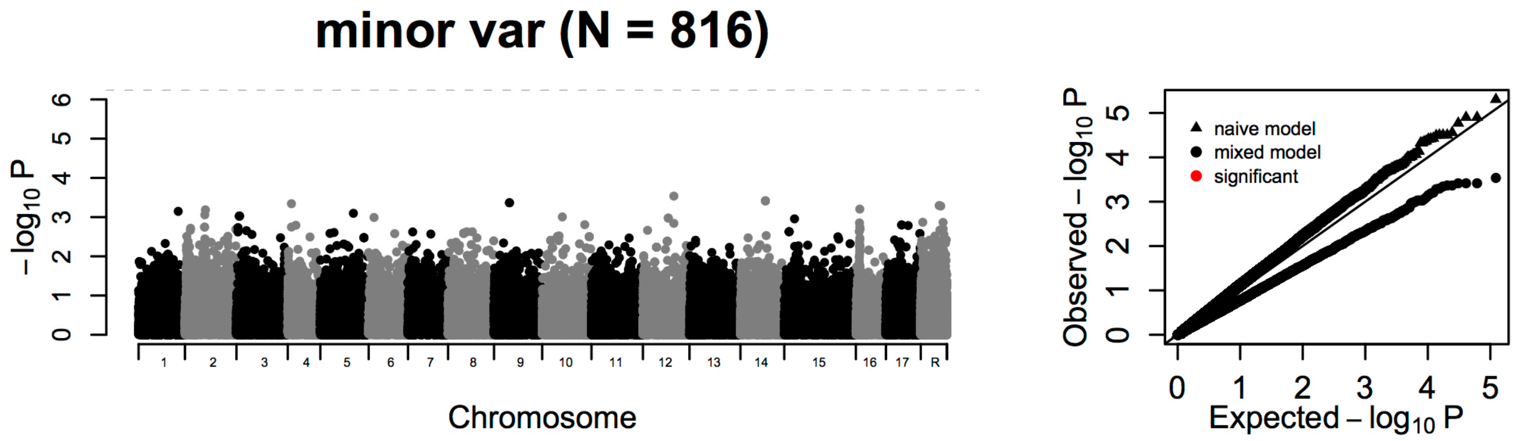

minor var (N = 816)
−log10 P
0 1 2 3 4 5 6
1 2 3 4 5 6 7 8 9 10 11 12 13 14 15 16 17 R
Chromosome
Observed − log10 P
Expected − log10 P
naive model
mixed model
significant

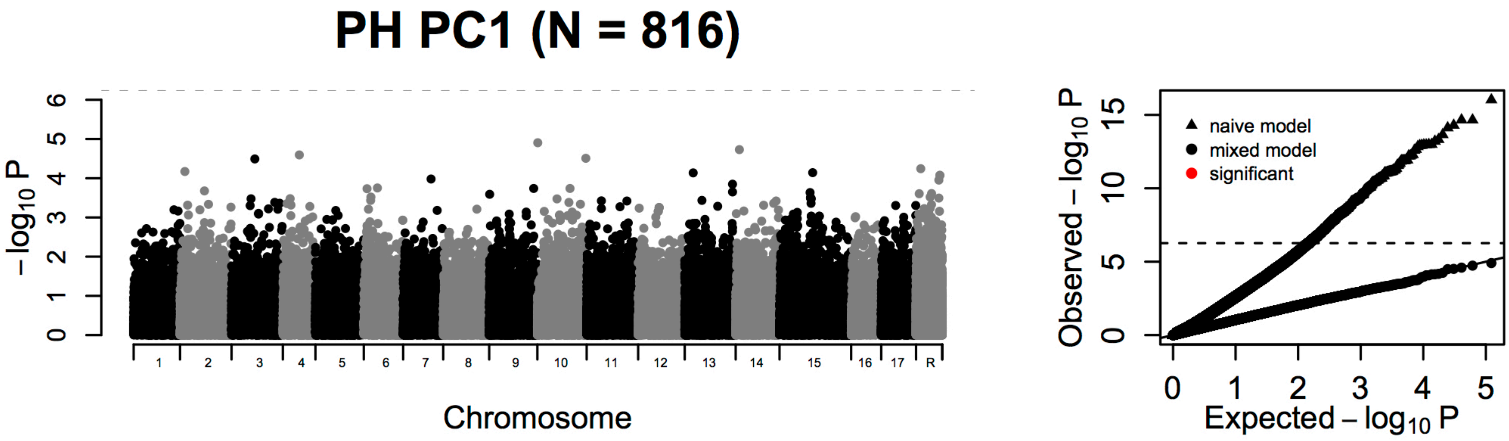
PH PC1 (N = 816)
– log10 P
Chromosome
1 2 3 4 5 6 7 8 9 10 11 12 13 14 15 16 17 R
Observed – log10 P
Expected – log10 P
naive model
mixed model
significant

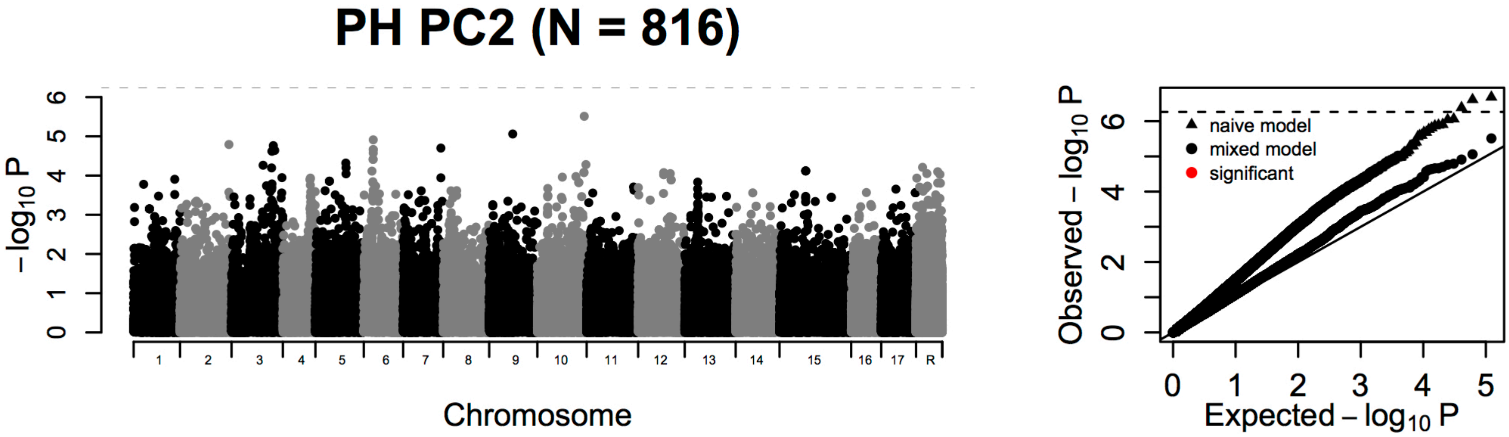
PH PC2 (N = 816)
– log10 P
Chromosome
1 2 3 4 5 6 7 8 9 10 11 12 13 14 15 16 17 R
Observed – log10 P
Expected – log10 P
naive model
mixed model
significant

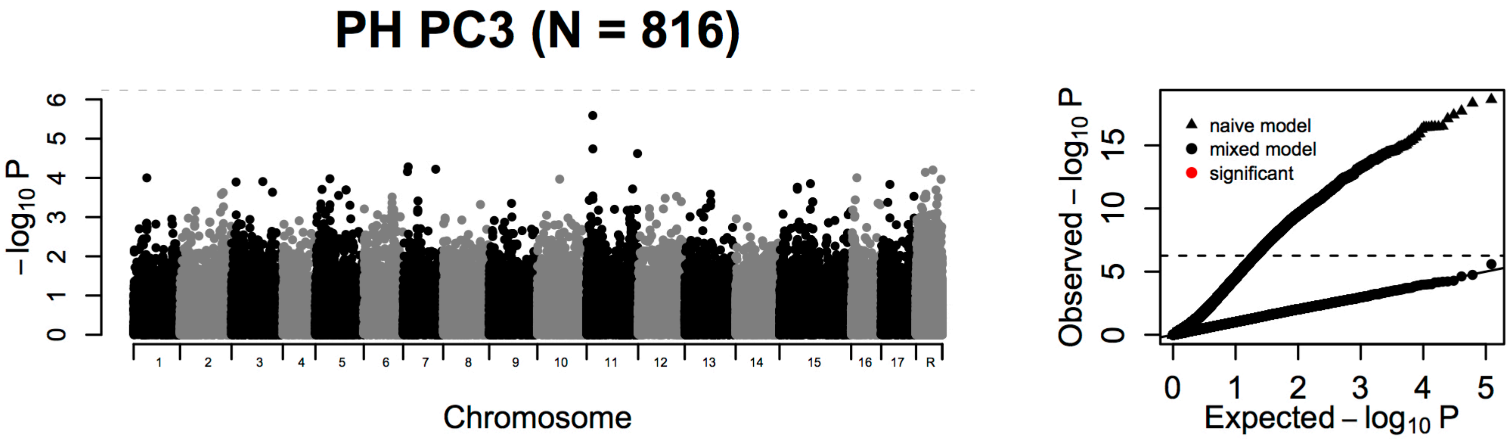
PH PC3 (N = 816)
– log10 P
Chromosome
1 2 3 4 5 6 7 8 9 10 11 12 13 14 15 16 17 R
Observed – log10 P
Expected – log10 P
naive model
mixed model
significant

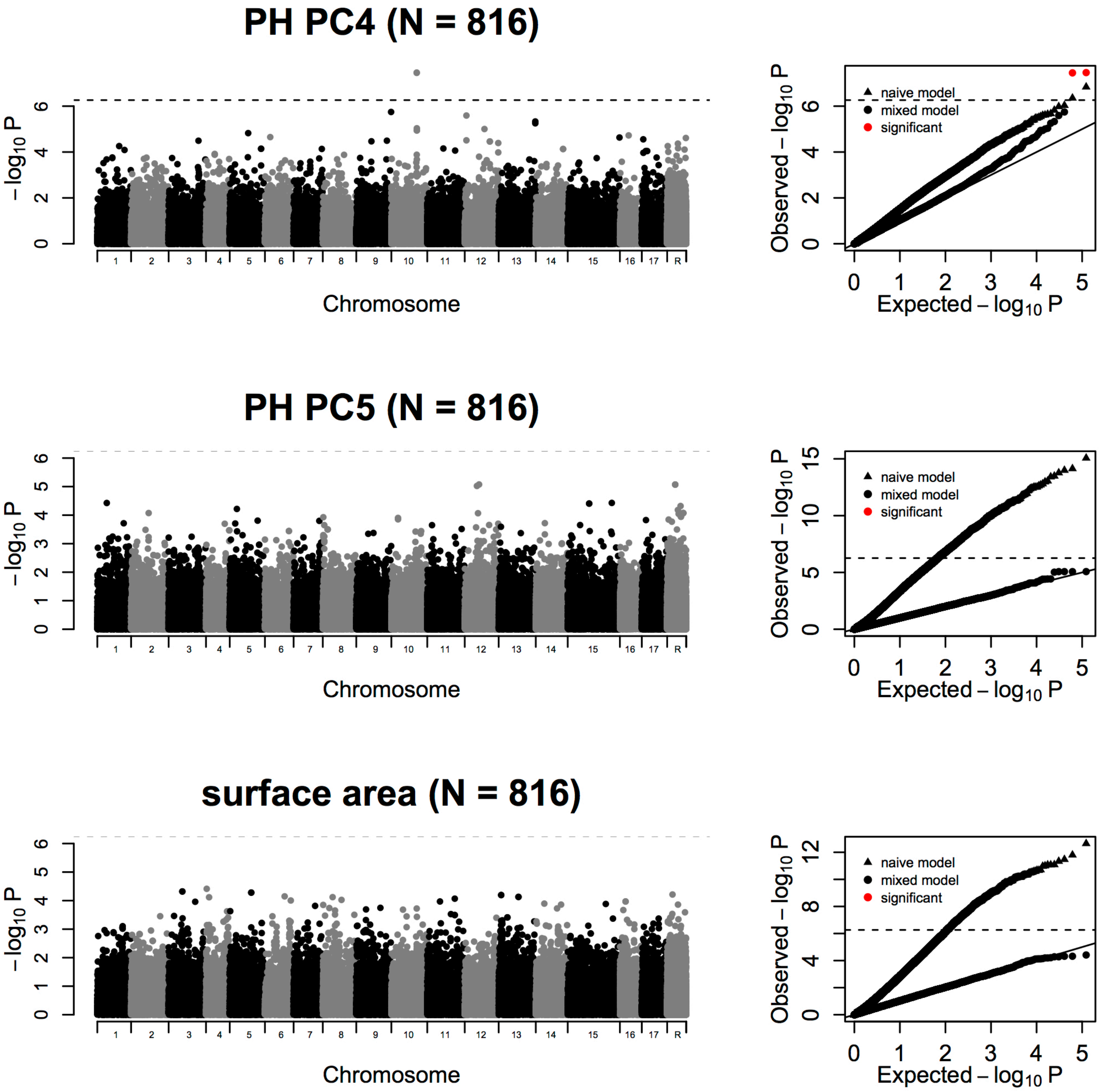

PH PC4 (N = 816)
−log10 P
Chromosome
Observed − log10 P
Expected − log10 P
naive model
mixed model
significant
PH PC5 (N = 816)
−log10 P
Chromosome
Observed − log10 P
Expected − log10 P
naive model
mixed model
significant
surface area (N = 816)
−log10 P
Chromosome
Observed − log10 P
Expected − log10 P
naive model
mixed model
significant

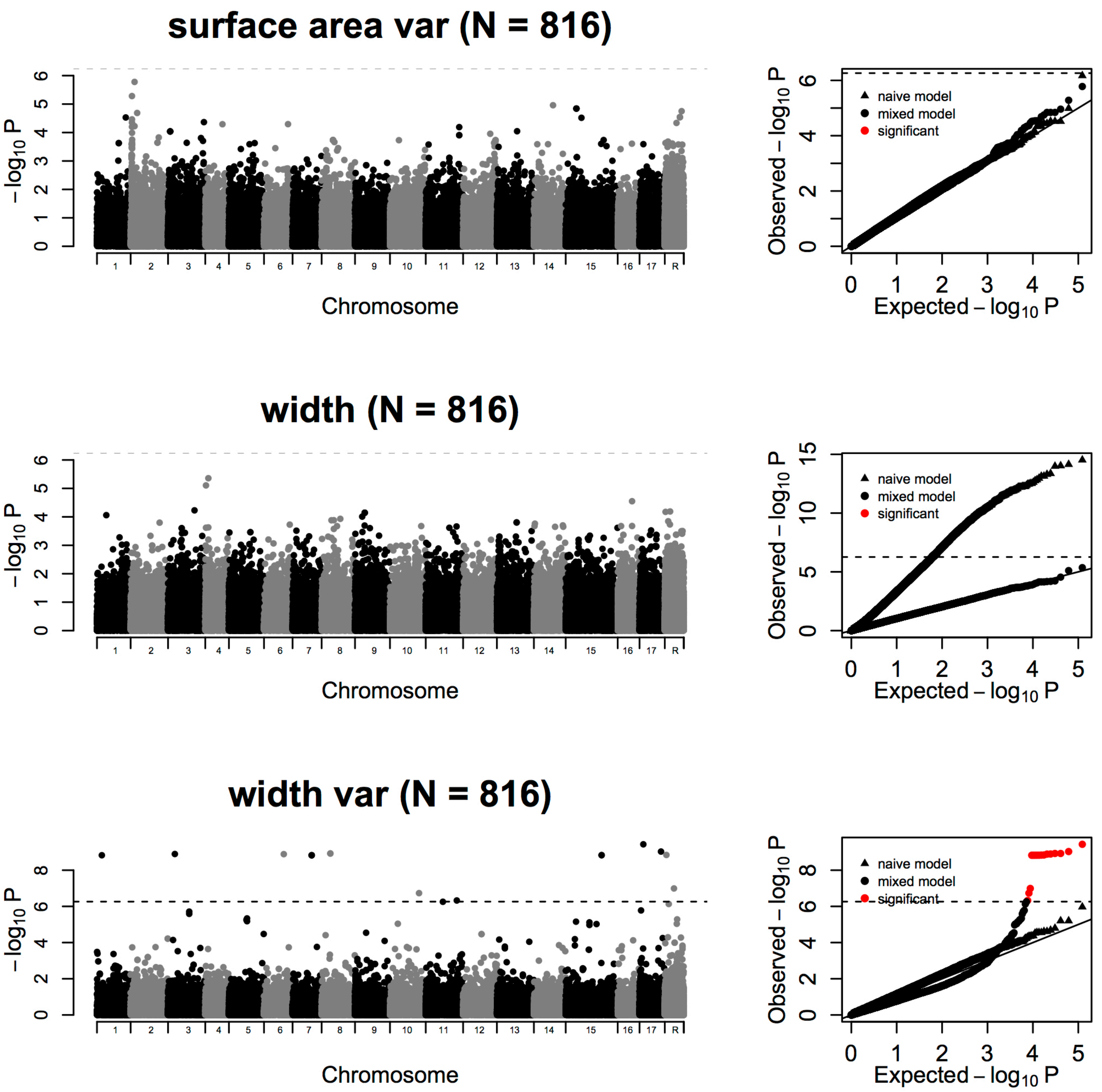
surface area var (N = 816)
−log10 P
Chromosome
1 2 3 4 5 6 7 8 9 10 11 12 13 14 15 16 17 R
Observed − log10 P
Expected − log10 P
naive model
mixed model
significant
width (N = 816)
−log10 P
Chromosome
1 2 3 4 5 6 7 8 9 10 11 12 13 14 15 16 17 R
Observed − log10 P
Expected − log10 P
naive model
mixed model
significant
width var (N = 816)
−log10 P
Chromosome
1 2 3 4 5 6 7 8 9 10 11 12 13 14 15 16 17 R
Observed − log10 P
Expected − log10 P
naive model
mixed model
significant
